# Cost of Living Dictates what Whales, Dolphins and Porpoises Eat: The Importance of Prey Quality on Predator Foraging Strategies

**DOI:** 10.1371/journal.pone.0050096

**Published:** 2012-11-21

**Authors:** Jérôme Spitz, Andrew W. Trites, Vanessa Becquet, Anik Brind'Amour, Yves Cherel, Robert Galois, Vincent Ridoux

**Affiliations:** 1 Littoral Environnement et Sociétés, UMR 7266, Université de La Rochelle/CNRS, La Rochelle, France; 2 Parc zoologique de La Flèche, La Flèche, France; 3 Marine Mammal Research Unit, Fisheries Centre, University of British Columbia, Vancouver, British Columbia, Canada; 4 Ifremer, Département Écologie et Modèles pour l’Halieutique, Nantes, France; 5 Centre d'Etudes Biologiques de Chizé, UPR 1934, CNRS, Villiers-en-Bois, France; 6 Observatoire PELAGIS - Systèmes d’Observation pour la Conservation des Mammifères et des Oiseaux Marins, UMS 3462 Université de La Rochelle/CNRS, La Rochelle, France; Hokkaido University, Japan

## Abstract

Understanding the mechanisms that drive prey selection is a major challenge in foraging ecology. Most studies of foraging strategies have focused on behavioural costs, and have generally failed to recognize that differences in the quality of prey may be as important to predators as the costs of acquisition. Here, we tested whether there is a relationship between the quality of diets (kJ·g^−1^) consumed by cetaceans in the North Atlantic and their metabolic costs of living as estimated by indicators of muscle performance (mitochondrial density, n = 60, and lipid content, n = 37). We found that the cost of living of 11 cetacean species is tightly coupled with the quality of prey they consume. This relationship between diet quality and cost of living appears to be independent of phylogeny and body size, and runs counter to predictions that stem from the well-known scaling relationships between mass and metabolic rates. Our finding suggests that the quality of prey rather than the sheer quantity of food is a major determinant of foraging strategies employed by predators to meet their specific energy requirements. This predator-specific dependence on food quality appears to reflect the evolution of ecological strategies at a species level, and has implications for risk assessment associated with the consequences of changing the quality and quantities of prey available to top predators in marine ecosystems.

## Introduction

The primary quest of animals is to obtain sufficient energy from food to maintain vital functions (i.e., basal metabolic rate) and support physiological and physical activities (e.g., costs of foraging, reproduction, and thermoregulation). Energy needed to fulfil the daily needs of animals can be considered an organism’s cost of living. It can be thought of as the energetic cost of sustaining a life, and varies by time of year, habitat, and life-history stage [1]–[3]. Such physical and biological differences mean that species with the same body masses may differ greatly in their respective costs of living despite the general relationship across the animal kingdom between body size (mass) and basal energy requirements [4]. Indeed, field metabolic rates of equivalent-sized homoeothermic species can differ by as much as 6 orders of magnitude [5].

In theory, the physiologies, morphologies and social systems of predators have been shaped by evolutionary processes that optimise prey capture or handling [6], [7]. The general acceptance that predators should attempt to maximize their energetic return during feeding events has resulted in energy fluxes becoming the primary underpinning of foraging theory frameworks [8] such as optimal foraging theory [9]–[11]. The primary tenet of optimal foraging theory is that natural selection should favour predators that maximize energy gained and minimize energy spent while foraging. Most foraging studies placed within this framework have focused on behavioural costs associated with handling time, pursuit time or the critical size of food patches [12]. They tend to recognize the importance that quantity of prey (i.e., biomass ingested per time unit) has on foraging behaviour, but generally overlook the importance that quality of prey (e.g., the energy acquired per unit of prey mass consumed) may play in influencing prey selection and ultimately determining the fitness of a predator. This shortcoming appears to be particularly true in marine ecosystems, where growing evidence suggests that the population dynamics of some species of top predators may be negatively affected when diets change from high-energy prey to lower-energy species [13], [14].

Marine mammals exhibit a large range of foraging strategies. Some are considered specialists, such as sirenians that graze on aquatic plants [15] or common dolphins (*Delphinus delphis*) that fulfil their high energy requirements with a diet mainly composed of caloric rich food [16]. However, small cetaceans and pinnipeds are most often described as opportunistic or generalist feeders with little or no feeding preferences. This categorization of their foraging strategies appears to be based on the taxonomic diversity of prey they consume, and fails to consider the functional characteristics of prey that are important to predators. It is thus commonly believed that marine mammals can thrive eating anything so long as there is sufficient biomass. Hence, fisheries models have tended to focus on the quantities of food consumed by marine mammals [17], but have generally failed to recognize that differences in the quality of prey may be as important to marine mammals as the sheer quantities of food.

Field observations and captive feeding studies suggest that some species of marine mammals may not be able to thrive on abundant low-energy prey whereas others may be less constrained by the quality of food they consume [14], [16]. Quantity cannot always replace quality [18], [19]. However, the extent to which this conclusion might be generalized and applied across all predator species remains untested despite the implication it holds for ecological theories and wildlife conservation.

We postulated that the costs of living for whales, dolphins and porpoises (cetaceans) should determine the quality of prey they consume, and tested whether such a relationship between diet quality and cost of living holds true across 11 phylogenetically–and ecologically–diverse species of cetaceans from the Northeast Atlantic Ocean. To this end, we used the mean energy density of prey recovered from stomachs as a proxy for diet quality, and used structural indicators of muscle performance (i.e., mitochondrial density and lipid content) measured from freshly dead animals as proxies for metabolic costs of living. We recognize that nutrient composition (e.g., amino acids, vitamins, etc.) also contribute to prey quality, but chose to only use energy density because it is readily available for most prey species and is a widely accepted metric of prey quality.

## Materials and Methods

### Diet Quality

Diets were determined from stomach content analysis reported in 32 studies for 3585 individual cetaceans feeding on 127 different prey species. We compiled the diet composition from published stomach content analyses for 11 species of cetaceans in the Northeast Atlantic belonging to 6 families (Balaenopteridae, Phocoenidae, Delphinidae, Ziphiidae, Physeteridae and Kogiidae): minke whale (*Balaenoptera acutorostrata*), fin whale (*Balaenoptera physalus*), harbour porpoise (*Phocoena phocoena*), common dolphin (*Delphinus delphis*), striped dolphin (*Stenella coeruleoalba*), bottlenose dolphin (*Tursiops truncatus*), long-finned pilot whale (*Globicephala melas*), Cuvier's beaked whale (*Ziphius cavirostris*), *Mesoplodon* beaked whale (*Mesoplodon* spp.), sperm whale (*Physeter macrocephalus*) and pygmy sperm whale (*Kogia breviceps*) [20]–[46]. Dietary data from stomach content analysis included prey species, and their numbers and mass, following standard analytical methods [30], [43], [47]. We obtained energy densities for 99 of the 127 prey species from proximate analyses. Energy densities were compiled for a wide range of marine forage species, including mesopelagic fish from the northeast Atlantic Ocean [48]. Additional data on energy densities for oceanic cephalopods were also used [49]. We then multiplied the ingested biomass by the energy density (kJ•g^−1^ wet mass) of each prey species consumed by each cetacean species to determine the mean energy value (quality) of a diet.

### Proxies for the Metabolic Cost of Living

We quantified the metabolic cost of living for each of the 11 species of cetaceans based on the structural characteristics of performance (i.e., mitochondrial density and lipid content) from muscle samples taken from 68 by-caught and beach-cast individuals (Table 1). All were adults in good nutritional status that had just died. Our rationale was that muscle tissue of active species uses O_2_ at a high rate, and is thus characterised by a high mitochondrial density and high lipid reserves. In contrast, phlegmatic predators have muscles with low O_2_ consumption and lower mitochondrial density and lower lipid reserves [50], [51].

**Table 1:** Sampling by cetacean species used for mitochondrial density and lipid content of the muscle.

	NUMBER OF INDIVIDUALS	
	Mitochondries	Lipids
Minke whale	6	5
Fin whale	8	4
Common dolphin	10	7
Striped dolphin	7	3
Bottlenose dolphin	7	5
Long-finned pilot whale	4	2
Harbour porpoise	5	4
Mesoplodon beaked whales	4	3
Cuvier's beaked whale	5	4
Pygmy sperm whale	2	-
Sperm whale	2	-

Standardized epaxial (swimming) muscle samples were collected from cetaceans by the French National Stranding Network along the Atlantic coasts of France between 2004 and 2010. Lipid extraction from muscle after freeze-drying and grinding followed standard analytical procedure [52], and total lipid content was measured with an Iatroscan after depositing concentrated aliquots of the lipid extracts onto Chromarods SII. To provide the mitochondrial density of muscle, we extracted total DNA from the same muscle samples using DNeasy Tissue kits (Qiagen). Amplification of DLOOP mitochondrial gene was then done by polymerase chain reaction using specific primers and a constant number of cycles for all samples. All PCR products and a standard dilution range were electrophoresed to determine the initial quantity of mitochondrial DNA in the muscle sample. Finally for each sample, we calculated mitochondrial density as the ratio between mitochondrial DNA and total DNA quantities.

### Data Analysis

We identified groups of cetaceans that had similar qualities of diets (energy densities) or metabolic costs of living (lipid content or mitochondrial densities of muscles) using Ward's hierarchical cluster analysis [53] calculated using the Euclidean dissimilarity coefficient of the species-individual matrix. The number of clusters representing the different classes of diet quality or costs of living was confirmed using non-parametric multiple pairwise comparison tests. We thus tested the among-species variability with non-parametric permutation-based one-way ANOVAs using species as a fixed factor. Permutation procedures were used if the residuals were not normally distributed, and multiple comparison tests (i.e., Conover-Inman non-parametric multiple pairwise comparison test) were conducted if interspecific differences were significant. Finally, we determined the significance of relationships between diet quality, cost of living and body mass using Pearson's correlation tests and linear models corrected for the non-independency of the error variance structure. All statistical analyses were performed with R software version 2.8.1. [54].

## Results

**Figure 1:**
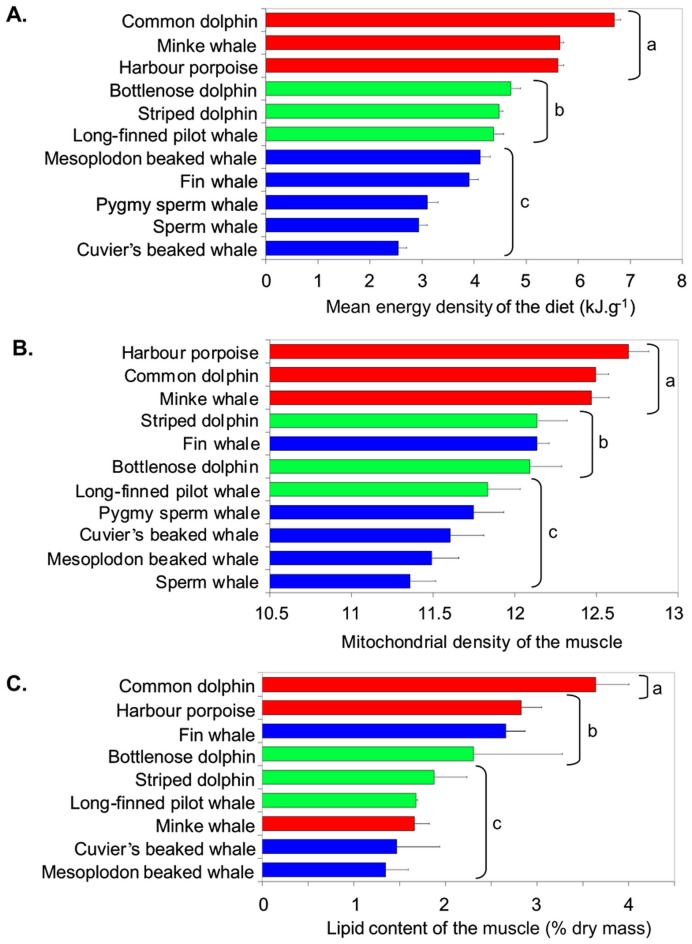
Differences between the quality of diets and the metabolic costs of living in cetaceans. Means ± s.d. with different letters (a, b, c) show significantly different groups of cetaceans. A: Mean energy density of the diets (kJ⋅g^−1^ wet mass). B: Cost of living as measured by the mitochondrial density of the muscle. C: Cost of living as measured by the lipid content of the muscle (% dry mass). Colours reflect the group of similarity identified for the mean energy density of diets: Group a in red, Group b in green and Group c in blue.

Mean energetic densities of the diet (MEDD) calculated for cetacean species and structural indicators of muscle performance showed a broad range of interspecific values and low intraspecific variations (Fig. 1). The mean energetic densities of the diet ranged from 1.7 to 7.2 kJ·g^−1^ (Fig. 1A). Three significant groups were distinguished by statistical analyses, (i) cetaceans with a high diet quality (MEDD>5.5 kJ·g^−1^) including common dolphin, harbour porpoise and minke whale (Group a Fig. 1A); (ii) cetaceans with a medium diet quality such as bottlenose dolphin, striped dolphin or long-finned pilot whale (Group b; Fig. 1A), and (iii) cetaceans with a low diet quality (MEDD<4.0 kJ·g^−1^) such as the sperm whale, pygmy sperm whale or Cuvier's beaked whale (Group c; Fig. 1A).

The mitochondrial densities of skeletal muscle showed a 15% relative difference measured between the species showing the lower mitochondrial density (the sperm whale) and the species with the highest mitochondrial density (the harbour porpoise) (Fig. 1B). Three significantly distinct groups were also identified, from cetaceans with high mitochondrial densities such as the common dolphin, harbour porpoise and minke whale (Group a; Fig. 1B), to cetaceans with low mitochondrial densities such as the sperm whale, pygmy sperm whale or Cuvier's beaked whale (Group c; Fig. 1B).

Total lipid content in muscle varied from 1.4 to 3.7% of the total dry muscle mass (Fig. 1C). Three significantly distinct groups were again identified: (i) the common dolphin that exhibited the highest values (>3%) (Group a; Fig. 1C), (ii) a group encompassing the bottlenose dolphin, harbour porpoise and fin whale that presented medium lipid contents (2–3%) (Group b; Fig. 1C), and (iii) other cetaceans, such as the beaked whales, which had lower lipid contents (<2%) (Group c; Fig. 1C). The two measures used as proxies for the metabolic cost of living–the mitochondrial density and the total lipid content–were based on muscle performance and were correlated (Pearson correlation test, *P*<0.005), confirming that these two structural characteristics co-vary positively within skeletal muscles of marine mammals.

**Figure 2:**
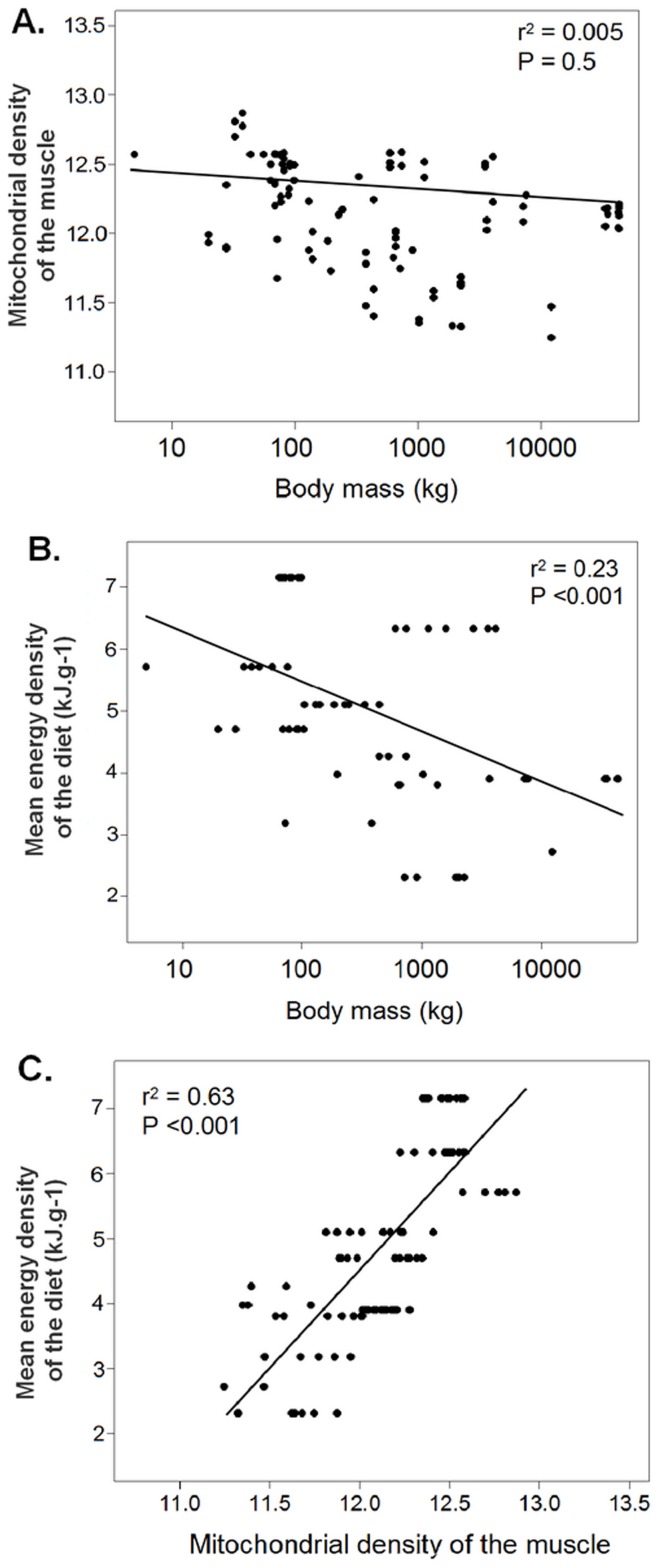
Relationships between body mass, the quality of diet and the metabolic cost of living in cetaceans. Cost of living is represented only by mitochondrial density of the muscle (lipid content of the muscle showed the same patterns but was measured for fewer species). Each data point represents a single individual. A: Mitochondrial density of the muscle as a function of log body mass (kg). B: Mean energy density of diets (kJ⋅g^−1^ wet mass) as a function of log body mass (kg). C: Mean energy density of diets as a function of mitochondrial density of the muscle.

In testing relationships between diet quality, body mass, and metabolic cost of living to better understand the dietary choices made by cetaceans, we found no relationship between body mass and cost of living (Pearson correlation test, *r* ^2^ = 0.113, *P*>0.05; Fig. 2A). There was a significant relationship between body mass and diet quality, but the explained variance was low (Pearson correlation test, *r* ^2^ = 0.228, *P*<0.001; Fig. 2B). The strongest relationship occurred between diet quality and the cost of living (Pearson correlation test, r^2^ = 0.633, *P*<0.001; Fig. 2C).

**Figure 3:**
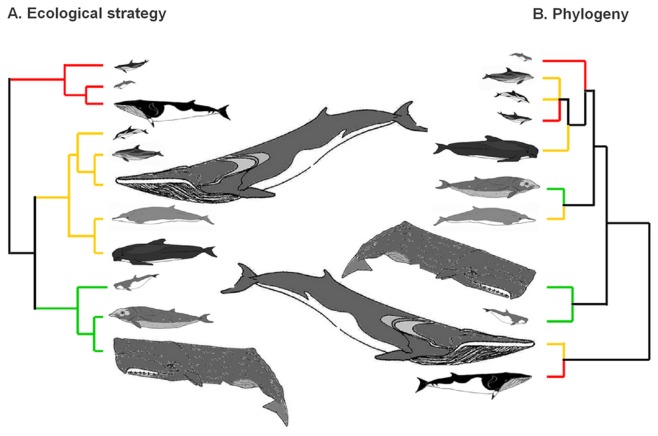
Branching diagrams showing the ecological and evolutionary relationships among cetaceans. Sizes of the cetaceans are shown to scale, and colours reflect whether the species have high (red), moderate (green) or low (blue) costs of living. A: The ecological strategy tree was produced using a cluster analysis of the three combined indicators (mitochondrial density of the muscle, lipid content of the muscle and diet quality). Species are arranged from highest (top) to lowest (bottom) costs of living. B: The actual cetacean phylogenetic tree [72]. Species are grouped by family from top to bottom into Phocoenidae (harbour porpoise); Delphinidae (bottlenose, striped and common dolphins, and long-finned pilot whale), Ziphiidae (Cuvier's beaked whale and Mesoplodon whales), Kogiidae (pygmy sperm whale), Physeteridae (sperm whale) and Balaenopteridae (minke and fin whales).

The cluster analysis of the three combined proxies (muscle mitochondrial density, muscle lipid content and diet quality) categorized cetaceans into three groups marked by different ecological strategies according to the quality of the prey they consumed (Fig. 3A). Species that fed on high quality foods with corresponding high metabolic costs of living included the common dolphin and harbour porpoise, while those that met their moderate cost of living with moderate quality foods included the bottlenose dolphin and fin whale. Species at the lowest end of the scale with low quality diets and low costs of living included the sperm whale and beaked-whales. This classification of ecological strategies did not appear to be strongly linked with individual body mass or phylogeny (Fig. 3)–as illustrated by common and striped dolphins that belong to the same family and are morphologically similar, but have different costs of living and different qualities of diets. These two dolphin species contrast sharply with the bottlenose dolphin and the fin whale that belong to different sub-Orders and are morphologically different, but have similar metabolic costs of living and similar qualities of diet (Fig. 3).

## Discussion

As hypothesised, cetaceans at the species level have diets of varying qualities and a wide range of costs of living. Our data show that a significant relationship exists for all cetaceans between diet quality and the metabolic cost of living from the smallest porpoise to the largest baleen whale. This relationship appears to be independent of body mass–and was not consistent with expectations stemming from the scaling relationship between body mass and metabolism [4], [55]. Our findings suggest that the variability among cetaceans in the qualities of their diets and their costs of living occurring at the species level are the consequences of ecological strategies shaped by evolutionary processes rather than physiological processes occurring at the phylogenetic or morphometric levels (Fig. 3).

Foraging on mobile prey requires predators to travel over large distances. It also requires the predators to use more energy to pursue, catch, kill and handle their targets. In contrast, foraging for food with no or low escape abilities reduces foraging costs [56]. Presumably, the costs of living dictate the minimum quality of food that each species of cetaceans must consume to survive. Cetaceans with higher metabolic costs of living must sustain themselves by targeting prey species with higher caloric densities, while species at the low end of the cost of living spectrum meet their needs by feeding on low quality food, and may not have the muscle performance needed to capture higher quality prey which are mainly pelagic, gregarious and highly mobile.

### Methodological Considerations

Cetaceans are large, long-lived and highly mobile species that forage underwater in large home ranges. They are difficult to observe in the wild, and many species are difficult or impossible to study in captivity. The limitations of obtaining biological samples from cetaceans [57], [58] means that diet described by stomach content analysis and structural characteristics of muscle measured from by-caught and beach-cast individuals are subject to uncertainty and biases that can affect perceptions of cetacean diets and their muscle characteristics. In spite of the well-known limitations of dietary analyses [59], [60], stomach content analysis is considered the best and most widely used method to quantify diets of top marine predators [47]. The independent published studies we used to describe the diet of each species were consistent with one another, and give us confidence that our descriptions of diet quality were reasonable for each species of cetacean in our study.

In terms of our measures of the metabolic costs of living, we recognized from the outset that mitochondrial densities and lipid contents in muscle can be affected by the origin of samples (i.e., carcasses of cetaceans). We therefore controlled our muscle sampling by avoiding sick individuals and selected only mature animals that were in good nutritional status and freshly killed (i.e., bycatch or beach-cast). This was made possible by the diversity of marine mammals in the Bay of Biscay, and the large spatial-temporal coverage of the French National Stranding Network that accumulated a large bank of tissues and maintained necropsy reports on sampled animals. Freezing and the causes of death were not deemed to have affected the mitochondrial density and lipid content of the muscle samples.

We took every precaution to limit known sources of bias and uncertainty in our sampling protocols and laboratory analyses. As such, our data reveal general patterns and relationships among quality of diets and the lipid content and mitochondrial densities of muscles that span a broad range of whales, dolphins and porpoise in the North Atlantic. Most notably, our data reveal a strong significant relationship between diet quality and the cost of living that supports the hypothesis that cetaceans select prey based on the quality of available species needed to meet their specific costs of living. Statistical analyses consistently identified three groupings of cetaceans based on similarities in the mean energy densities of their diets, the mitochondrial densities of their muscles, and the proportions of lipid in their muscles (Fig. 1). Of the three groups, the third one (Group c) that had the poorest quality diets and lowest costs of living had higher variation between individuals than the other two groups. A larger sample size for some of species in this third group would likely reduce this variability and give the group greater coherence.

### Implications on Ecological and Physiological Theories

The “food-habit hypothesis” [61] and the “muscle performance hypothesis” [56] are two controversial theories that have been proposed to explain the relative effects observed in experimental studies of diet on basal or field metabolic rates [5], [61], [62]. However, we suggest that attempts to draw such meaningful associations between the metabolism and qualitative dietary assessments have generally fallen short because energy density (kJ·g^−1^) rather than food type (e.g., vertebrates *versus* invertebrates, or animals *versus* plants) appears to be a more meaningful way to quantify diet quality. Protein content, vitamins and micronutrients compositions or digestibility are other measures of dietary quality, but energy density offers a more robust and standard quantitative proxy of diet quality that can be easily measured on a wide range of food items.

The relationship between cost of living and diet quality can be understood on different time scales. On an evolutionary time scale, predator species that developed foraging strategies targeting mobile prey would likely have increased their muscular performance–and their basal and field metabolic rates in turn–to capture prey compared to predators foraging on species with little or no escape abilities [56]. However, on an ecological time scale, we propose that the predator costs of living reflect and dictate the quality of foods they consume, and that predator metabolic rates are not driven by the quality of prey they consume. Thus, species with higher costs of living must fulfil their energy requirements by targeting prey species with higher caloric densities, while species with lower costs need only to feed on low quality prey to thrive. Consequently, the general relationship we found between diet quality and the cost of living should apply to more species than just whales, dolphins and porpoises. The quality of diet framework we propose to understand the foraging ecology of cetaceans should thus apply equally well to understanding the dietary choices and needs of other animals such as birds, small terrestrial mammals or reptiles [5], [63], [64].

Our findings are consistent with the optimal foraging theory prediction that predators should prefer prey that yield more energy than the energy expended to obtain it [12], [65]. While this prediction has generally been inferred to imply optimization of behavioural costs of foraging, we propose that it may also reflect optimization of energy intake (to meet fixed costs of living) relative to the stomach capacity of the predator. Hence, predators must have an adaptable foraging strategy in terms of foraging behaviour and functional prey selection that is directly related to their specific energy requirements.

Although we reason that foraging strategies are largely influenced by the metabolic costs of living, we recognize that life history and ontogeny also contribute to the ultimate cost of living such that species can differentially allocate energy intake towards growth, survival or reproduction [66], [67]. Specific reproductive strategies may also modify seasonally energy needs of some species depending on the duration of the reproductive period or parental care [68], [69]. All such demands impact the cost of living, and are best sustained by an increase in food quality. In our study, we limited the effect of life history on the cost of living within species by only studying adults with similar reproductive strategies and status (e.g., no pregnant or lactating females). Future studies that incorporate life-history variability (e.g., inter-birth interval or migration patterns) may further explain some of the variability we observed in the relationship between muscle performance and diet quality.

### Implications on Wildlife Conservation

Marine biodiversity is being widely affected by climatic shifts and the human impacts of global warming and fishing [70], [71]. A number of ecosystems are seeing the emergence of junk-food as biodiversity is perturbed and ecosystems shift from high-quality species (i.e., species with high energy densities per mass unit) to low-quality species [13]. Consequently, the population dynamics of species with high costs of living (such as some species of marine birds and mammals) may be negatively affected by the increased abundance of junk-food in marine ecosystems [13].

The relationship between diet quality and cost of living alters current understanding of the foraging ecology of top marine predators and has bearing on wildlife conservation. Our results suggest that the risk to cetaceans faced with changes in the quality and quantity of prey available to them varies among cetaceans species and is closely linked to the costs of living of each species. Hence, the sensitivity of cetaceans to changes in the prey available to them will be higher for those species that have higher costs of living than they will be for species of cetaceans that thrive on low quality diets. This is because predators that can thrive on low quality food are likely to have more options than those that must meet their nutritional requirements with the higher quality prey species. A classification of cetaceans based on the three energetic categories we identified could be used to prioritize monitoring and management efforts at a species level within marine ecosystems subjected to human exploitation and global changes.

### Conclusions

Major insights into the ecology and physiology of animals that are difficult to study (either because they are too large to be handled, or are cryptic, protected, or even extinct) can likely be obtained by quantifying the quality of their diets (in terms of energy content) and deriving proxies for their costs of living (in terms of muscle performance). Our application of this approach suggests that the costs of living dictate the quality of food that cetaceans must consume to survive. This relationship is consistent with ecological expectations associated with the cost of living and the co-evolution of predator-prey relationships, but is inconsistent with phylogenetic and body mass expectations. We believe our study is a first step towards developing an energetically-based unifying theory about prey-predator relationship that may help to better understand the ecology of predators and guide future wildlife conservation.
